# Malignant isolated cortical vein thrombosis with type II protein S deficiency: a case report

**DOI:** 10.1186/s12883-016-0597-0

**Published:** 2016-05-18

**Authors:** Nobuhiko Arai, Masanao Tabuse, Akiyoshi Nakamura, Hiromichi Miyazaki

**Affiliations:** Department of Neurosurgery, Hiratsuka City Hospital, 1-19-1 Minamihara, Hiratsuka, Kanagawa 254 0065 Japan

**Keywords:** Pregnancy, Type II protein S deficiency, Isolated cortical vein thrombosis, Computed tomography venography, Magnetic resonance venography

## Abstract

**Background:**

The incidence of cerebral venous thrombosis (CVT) is low, and in particular, isolated cortical vein thrombosis (ICVT) is very rare. The diagnosis of ICVT is difficult by using conventional computed tomography (CT) and magnetic resonance imaging (MRI). However, with appropriate treatment, ICVT has a good prognosis.

**Case presentation:**

Herein, we present a rare case of a 40-year-old woman with ICVT and type II protein S (PS) deficiency, who experienced a stroke. She initially presented with generalized convulsions. A CT scan showed intracerebral hemorrhage (ICH) in the left temporoparietal region. However, her condition rapidly deteriorated and she went into a coma approximately 20 h after admission. A second CT scan revealed significant ICH expansion and transfalcine herniation. Decompressive hemicraniectomy with duraplasty was performed, and ICVT was confirmed owing to abnormal vascular tone and black appearance of the cortical vein. She underwent anticoagulation therapy and rehabilitation, and gradually recovered.

**Conclusion:**

We experienced an extremely rare case of isolated cortical vein thrombosis related with type II PS deficiency. CT-digital subtraction angiography is a useful supportive technique in the diagnosis of ICVT. Decompressive hemicraniectomy is effective for hemorrhage extension cases, and ICVT with hemorrhage might require early anticoagulation therapy.

## Background

Cerebral venous thrombosis (CVT) is a relatively rare cause of stroke, and it has been reported to cause 0.5–1.0 % of all strokes [1, 2]. The International Study on Cerebral Venous and Dural Sinuses Thrombosis (ISCVT) reported that approximately 17 % of stroke cases involved cortical vein occlusions [3]. CVT without occlusion of the major dural venous sinuses or deep cerebral veins is termed isolated cortical vein thrombosis (ICVT) [4]. Headache is less common in ICVT (71 %) than in major sinus thrombosis (89 %) [5]. Papilledema has not been reported previously. Parenchymal brain lesions have been found to be more common in ICVT cases than in sinus thrombosis cases (81 % vs. 63 %) [3]. For diagnosis, magnetic resonance imaging (MRI) is the most frequently used method. However, in some cases, it is difficult to detect a lesion on conventional MRI, and in these cases, angiography is necessary. Anticoagulation therapy appears to be the standard therapy in patients with ICVT without hemorrhage. However, it is controversial whether anticoagulation therapy should be used in ICVT cases with intracerebral hemorrhage (ICH). When ICH extends and impending herniation occurs very infrequently, decompressive cranioctomy should be performed; however, this is not the standard therapy [6]. If CVT without ICH is diagnosed in the early stage and appropriate therapy is administered, the prognosis is excellent, and this applies to ICVT. Here, we present an extremely rare case of a patient with ICVT and type II Protein S (PS) deficiency, who experienced exacerbation without anticoagulation therapy.

## Case presentation

A 40-year-old woman was admitted to our hospital because of generalized convulsions. She delivered her first child 3 weeks ago and was asymptomatic until the seizure onset. She had no medical history, including eclampsia, and no family history of hypercoagulable states. Her seizures were the generalized type and stopped spontaneously after 2 minutes. She denied experiencing headache, and no papilledema was noted.

**Fig. 1:**
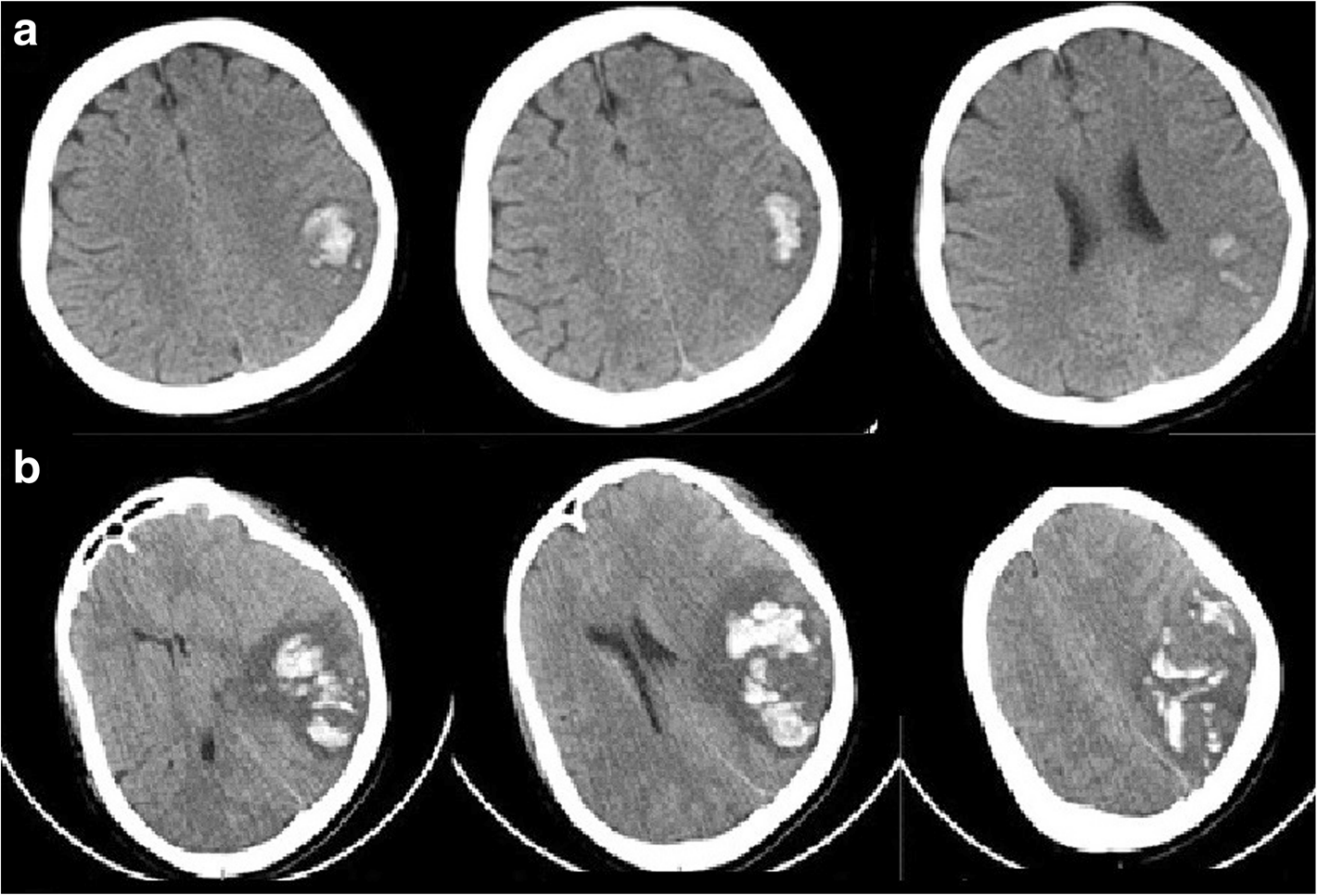
**a** Computed tomography images showing intracerebral hemorrhage in the left temporoparietal lobe, without brain midline shift before exacerbation. **b** Computed tomography images showing intracerebral hemorrhage expansion in the temporoparietal lobe and brain midline shift after exacerbation

**Fig. 2:**
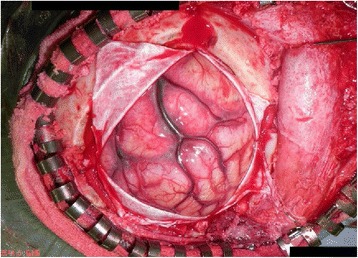
An intraoperative image showing a black and stiff cortical vein and congestion of the cerebral surface with the occlusive vein

**Fig. 3:**
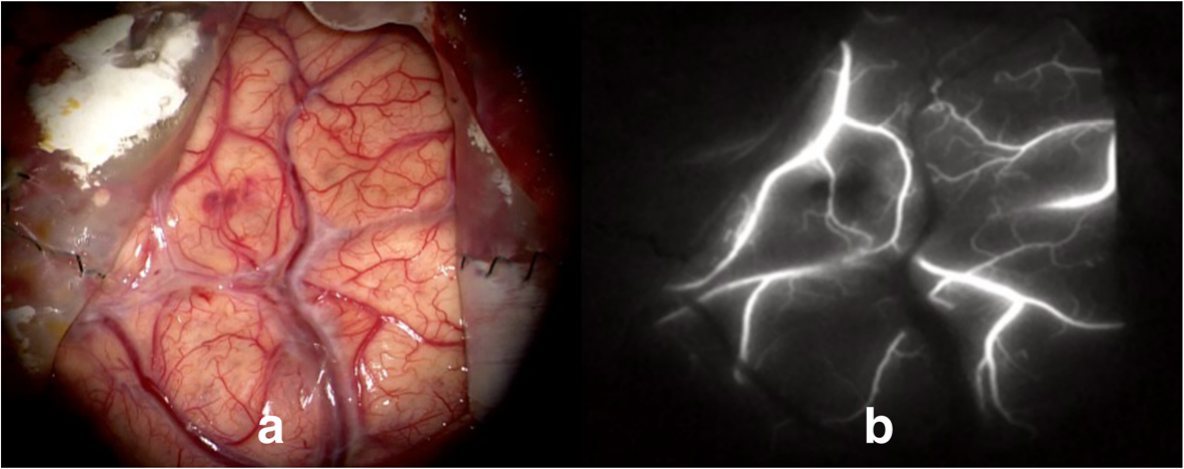
**a** An intraoperative image showing a thin and shrunken cortical vein. **b** An indocyanine green (ICG) angiography image showing absence of blood flow in the cortical vein

On admission, her blood pressure (BP), heart rate, and body temperature were 138/88 mmHg, 126 beats/min, and 37 °C, respectively. Neurological deficits were not identified. A CT scan revealed ICH in the left temporoparietal lobe (Fig. 1a). Considering the age of the patient and absence of a hypertension history, CT-digital subtraction angiography (CT-DSA) was performed to rule out conditions such as an aneurysm, arteriovenous malformation (AVM), and sinus thrombosis, and no such conditions were noted. The coagulation profile was assessed, and blood examinations were performed to check for connective tissue diseases. No abnormal data was found, except that the PS activity was low at 37 % (Table 1). This low level was thought to have resulted from her recent pregnancy state. Her ICH was treated with conservative medical therapy in order to maintain her BP below 120 mmHg. MRI and cerebral angiography were planned on another day to identify the definite etiology. However, 20 hours after admission, her response to stimuli suddenly decreased, and she presented with right hemi-paralysis. She did not experience seizures or headache at that time. Her conscious level was Glasgow Coma Scale (GCS) E2V3M5. A CT scan showed ICH expansion and brain midline shift (Fig. 1b). Therefore, emergent external decompression with duraplasty was performed. Intraoperatively, the cortical vein appeared black and stiff, and the cerebral surface with the occlusive vein was congested (Fig. 2), indicating possible thrombosis of the cortical vein. After the operation, her intracranial pressure (ICP) was controlled using glycerols and deep sedation with propofol. An ICP monitor was not used. For the ICVT, systemic heparinization was started. MRI/MRA after the operation showed no right white matter edema or narrow major vessels, which lowered the possibility of a differential diagnosis of posterior reversible encephalopathy syndrome (PRES). The PS amount and PS activity were assessed again 10 weeks after delivery. The PS amount was found to be normal; however, the PS activity was still below normal (Table 1). Therefore, she was diagnosed with type II PS deficiency. On the 35th postoperative day (POD), her brain swelling reduced, and cranioplasty was performed. Indocyanine green (ICG) angiography indicated occlusion of the cortical vein (Fig. 3). She underwent rehabilitation and was discharged from the hospital on the 58th POD. On discharge, her GCS was E4V5M6 and modified Rankin scale score was 3.

**Table 1:** Protein S (PS) and protein C (PC) values in our patient

	After labor			Normal range
	3 weeks	6 weeks	10 weeks	
PC activity (%)	59	100	114	70–150
PS activity (%)	37	35	46	60–127
PS amount (%)	N/A	77	97	65–135
Free PS amount (%)	N/A	54	86	60–150

*N/A* not applicable

## Discussion

**Fig. 4:**
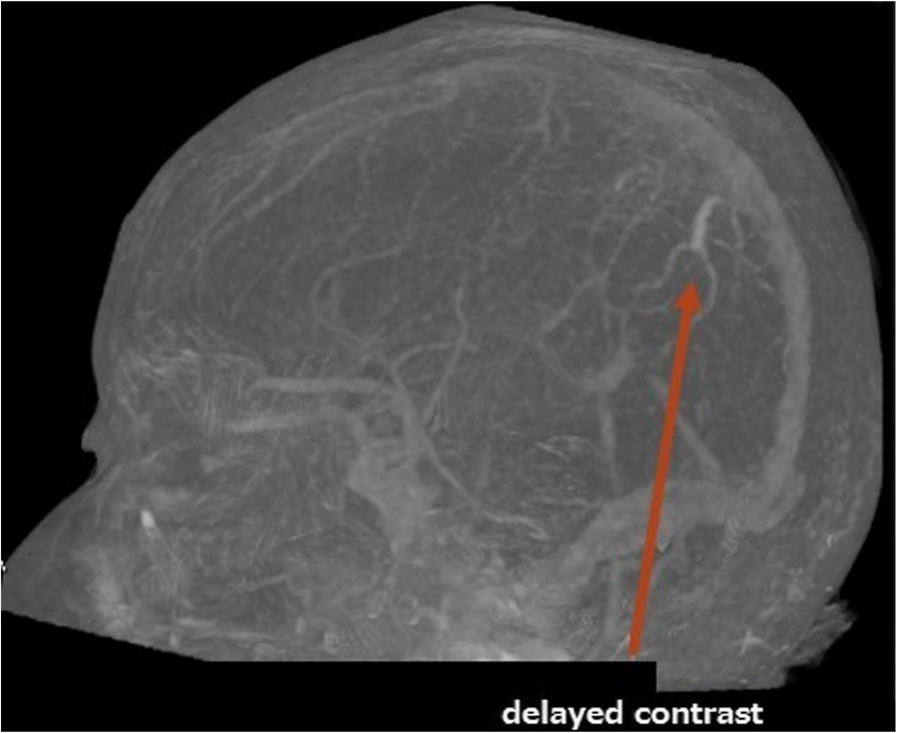
A time delay computed tomography-digital subtraction angiography image obtained at admission showing the culprit cortical vein. This vein coincided with the occluded cortical vein shown in the intraoperative image (Fig. 2)

CVT is a relatively rare cause of stroke and has been reported to cause 0.5–1.0 % of all strokes [1, 2]. The incidence of ICVT is much lower than that of CVT. There are no specific symptoms for CVT. The diagnosis is very difficult owing to the rarity of the condition and obscurity of symptoms. In the meantime, it is helpful to note a patient’s background and history. The risk factors that can be easily identified by history taking include pregnancy, puerperium, known connective tissue disease, known malignancy, contraceptive use, and infections, such as sinusitis, otitis, and mastoiditis [7, 8]. Young individuals, especially young women are very vulnerable to this disease. The ISCVT reported that 78 % of all CVT cases were aged <50 years and that the incidence of CVT was 3 times greater in women than in men [3]. If a young individual, particularly a pregnant or puerperal woman, presents with new onset stroke-like symptoms such as headache and seizure, it is important to check for the presence of the above-mentioned risk factors for CVT. If CVT is suspected, CT venography (CTV) or MR venography (MRV) should be performed. However, ICVT is hard to detect using these conventional modalities because of its small size. A recent report showed that the T2*/susceptibility-weighted image (SWI) MR sequence is very effective to detect lesions [9]. In our case, because we suspected CVT but not ICVT, only CTV was checked and all major sinuses appeared patent. If we had checked the SWI, the lesion might have been detected and early intervention could have been performed. If the initial CT-DSA is retrospectively checked thoroughly, suspecting any cortical vein lesion, contrast material in the culprit cortical vein can be identified (Fig. 4). This showed that only a CT-DSA image would be sufficient for the diagnosis of IVCT, and that MRI (SWI) or angiography may not be imperative in some cases.

Anticoagulation for CVT prevents expansion of the thrombus and allows for recanalization of the occlusion site. This therapy is supported by the guidelines of the European Federation of Neurological Societies (EFNS). However, in case of hemorrhage presentations, appropriate therapy is very controversial. It has been reported that 39–41 % of CVT patients presented with ICH, hemorrhagic venous infarcts, or isolated subarachnoid hemorrhage [10]. Anticoagulation therapy might worsen the condition, and its use is therefore controversial. Some reports suggest that the prognosis of ICVT is favorable without anticoagulants [11, 12]. However, without anticoagulation therapy, our patient’s condition worsened within 24 h even though the lesion was only an isolated CVT. This event possibly suggests the importance of immediate anticoagulation therapy for not only major sinus occlusions but also ICVT, which is also asymptomatic or incidental. Additionally, if the ICH expands despite the use of these therapies and contributes to impaired consciousness, decompressive hemicranioctomy appears to be appropriate to control ICP, as in our case [6].

PS is a vitamin K-dependent anticoagulant and its deficiency is thought to be major risk factor for CVT. According to the classification system proposed in Munchen in 1992, PS deficiency is classified as type I (low total and free antigen, reduced activity), type II (normal total and free antigen, reduced activity), and type III (normal total antigen, reduced free antigen and activity). Our patient was diagnosed with type II PS deficiency, which is an extremely rare disease. Although the prevalence of PS deficiency in Japan is estimated to be more than that in European countries, PS deficiency has been estimated at less than 0.5 % in the general European population [13]. Type I and type III PS deficiencies have been reported to account for almost all cases of PS deficiency [14]. Therefore, type II PS deficiency is considered very rare. When PS is suspected as a risk factor of CVT in pregnant or puerperal women, the time at which PS is measured after labor is important. A previous study reported that the free PS levels decreased significantly from the first trimester to the second trimester [15]. Another study reported that the PS activity in the third trimester reduced to 16–42 % [16]. The time when PS activity returns to normal in the postpartum period is unclear. However, it is believed that the PS activity normalizes at approximately 1 month after delivery. Therefore, as in our case, it is important to check PS at least 6 weeks after delivery. PS deficiency can be misdiagnosed as conditions, such as vitamin K deficiency, liver disease, nephritic syndrome, and disseminated intravascular syndrome. Therefore, these conditions should also be excluded. We experienced an extremely rare case of type II PS deficiency, which contributed to the occurrence of ICVT.

## Conclusion

Here, we presented an extremely rare case of a patient with ICVT and type II PS deficiency, who experienced a stroke. The patient was successfully treated with external decompression and anticoagulants. Venous thrombosis and CVT should be considered in pregnant or puerperal women who experience stroke. For early diagnosis, it is vital to suspect CVT, including ICVT, considering the patient’s background. In such a case, CT-DSA should be performed and the images should be thoroughly checked. A hematoma associated with ICVT caused by a cortical vein might expand without early anticoagulation therapy, as was noted in our case. Therefore, early anticoagulation therapy might be essential, even in isolated cases involving ICH.

### Ethics statement

This case report involves a patient. The case report complies with the Helsinki Declaration, and approval was obtained from the ethics committee of Hiratsuka City Hospital.

### Consent for publication

Written informed consent was obtained from the patient for publication of this case report and any accompanying images. A copy of the written consent is available for review by the Editor of this journal.

### Availability of data and materials section

The datasets supporting the conclusions of this article are included within the article and its additional files.

## Abbreviations

AVM: arteriovenous malformation
BP: blood pressure
CT: computed tomography
CT-DSA: CT-digital subtraction angiography
CTV: CT venography
CVT: cerebral vein thrombosis
EFNS: European Federation of Neurological Societies
GCS: Glasgow Coma Scale
ICG: Indocyanine green
ICH: intracerebral
ICP: intracranial pressure
ICVT: isolated cortical vein thrombosis
ISCVT: International Study on Cerebral Venous and Dural Sinuses Thrombosis
MRI: magnetic resonance imaging
MRV: MR venography
PS: protein S
SWI: susceptibility weighted image
